# NSAID-Induced Acute Interstitial Nephritis Concurrent With IgA Vasculitis: A Case-Based Systematic Review

**DOI:** 10.1016/j.xkme.2026.101462

**Published:** 2026-07-10

**Authors:** Carmela Caputo, Concetto Sessa, Vittorio Serio, Federica Baciga, Luigi Annicchiarico Petruzzelli, Bruno Minale, Walter Morale, Alessandro Mantovani, Antonio Granata, Louise Oni, Matko Marlais, Gabriele Malgieri, Rukshana Shroff, Yuri Battaglia

**Affiliations:** 1 Pediatric Nephrology and Dialysis; Kidney Transplant Center, AORN Santobono Pausilipon, Naples, Italy; 2 Nephrology and Dialysis Department, “Maggiore” Hospital, Modica, Ragusa, Italy; 3 Department of Medicine, University of Verona, Verona, Italy; 4 Nephrology and Dialysis Unit, Pederzoli Hospital, Peschiera del Garda, Verona, Italy; 5 Metabolic Diseases Research Unit, IRCCS Sacro Cuore-Don Calabria Hospital, Negrar di Valpolicella (VR), Italy; 6 Nephrology and Dialysis Unit, “Cannizzaro” Emergency Hospital, Catania, Italy; 7 Department of Women's and Children’s Health, University of Liverpool, Liverpool, UK; 8 Department of Paediatric Nephrology, Great Ormond Street Hospital for Children, London, UK; 9 Renal Unit, UCL Great Ormond Street Hospital and Institute of Child Health, London, UK

**Keywords:** hematuria, Henoch-Schönlein purpura, kidney biopsy, nonsteroidal anti-inflammatory drugs, proteinuria

## Abstract

Overlap between acute interstitial nephritis (AIN) and glomerulonephritis is uncommon and diagnostically challenging. We present the first pediatric case of clinically diagnosed immunoglobulin A vasculitis nephritis (IgAV-N) concurrent with nonsteroidal anti-inflammatory drug (NSAID)-induced AIN. A 14-year-old boy was hospitalized for recurrent gastroenteritis, purpura, and arthralgia treated with ibuprofen. After 5 days, he developed stage 3 acute kidney injury, subnephrotic proteinuria, hypertension, and oligoanuria. Kidney biopsy showed mild to moderate interstitial inflammation with tubulitis; immunofluorescence was negative for glomerular IgA, with nonspecific tubular C3 staining. After NSAID discontinuation, 10 dialysis sessions, and corticosteroids, kidney function recovered completely within 3 months. To support the clinical lesson highlighted by our case, we conducted a systematic review of biopsy-confirmed AIN–glomerulonephritis overlap. PubMed, Scopus, Web of Science, and Google Scholar were searched for biopsy-confirmed AIN with concurrent glomerulonephritis. Clinical features, etiologies, treatments, and outcomes were extracted. Systematic review identified 8 studies (12 patients). Drug-related AIN predominated (9 of 12), followed by infection (2 of 12) and autoimmune disease (1 of 12). Nephrotic-range proteinuria was described in 6, hematuria in 8, and hypersensitivity findings in 7 of the 12 patients. Complete recovery occurred in 7 and partial recovery in 3 patients, and dialysis was required in 2 of the 12 patients. NSAID-induced AIN concurrent with IgA vasculitis nephritis should be suspected in atypical acute kidney injury.

## Introduction

Immunoglobulin A vasculitis nephritis (IgAV-N) or Henoch-Schönlein purpura is the most common systemic vasculitis in children.^1^ The incidence is 3-27 cases/100,000 children per year^2^ and is more frequent in male patients.^3^ IgAV-N is clinically characterized by palpable purpura, arthritis, gastrointestinal symptoms, and kidney involvement.^4^ Kidney involvement occurs in 20%-54% of pediatric cases.^1^ However, acute kidney injury (AKI) is rarely reported as the initial presentation of kidney damage in IgAV-N.^5^

Although pediatric cases of IgAV-N overlapping with other kidney pathologies have been documented,^6^^,^^7^ to our knowledge, no published cases describe the coexistence of IgAV-N and acute interstitial nephritis (AIN) induced by nonsteroidal anti-inflammatory drugs (NSAIDs) (Table S1). This case report presents a child with IgAV-N complicated by NSAID-induced AIN and explores this rare and clinically significant diagnostic overlap through a systematic literature review.

## Methods

The study was conducted according to the 1995 Declaration of Helsinki and its revisions. Methodological details are provided in the Supplementary Material (Item S1: Methods and Tables S2-S4). The case report was reported according to CARE guidelines.

## Results

### Case Report

In February 2025, a previously healthy 14-year-old boy was hospitalized in the pediatric department of his local hospital after recurrent episodes of gastroenteritis and ankle arthralgia. On clinical examination, reticular purpuric lesions confluent over the lower limbs were observed, with peripheral edema limited to the areas of purpura (Fig S1). Blood chemistry showed serum creatinine concentration 0.6 mg/dL with estimated glomerular filtration rate >90 mL/min by the CKiDU25 (Chronic Kidney Disease in Children Under 25) equation,^8^ increased C-reactive protein at 45 mg/L (reference range <3.5 mg/L), and hypocomplementemia (C3 0.79 g/L [reference range 0.90-1.8 g/L], C4 0.04 g/L [reference range 0.10-0.40 g/L]) (Table S5). Urinalysis showed albumin (>4+ on dipstick) and microhematuria; the urinary sediment contained red blood cells, leukocytes, and hyaline casts (Table S6). A throat swab was positive for *Streptococcus pyogenes*. For pain control, the patient was prescribed ibuprofen at a dose of 600 mg 3 times daily for 5 days (recommended pediatric dosing for adolescents aged 12-18 years: 20-40 mg/kg/d).

On hospital day 5, while receiving ibuprofen, the patient developed stage 3 AKI (serum creatinine 2.7 mg/dL; serum urea 107 mg/dL), accompanied by arterial hypertension and oligoanuria. He was transferred to a specialist pediatric nephrology team for further management.

**Figure 1:**
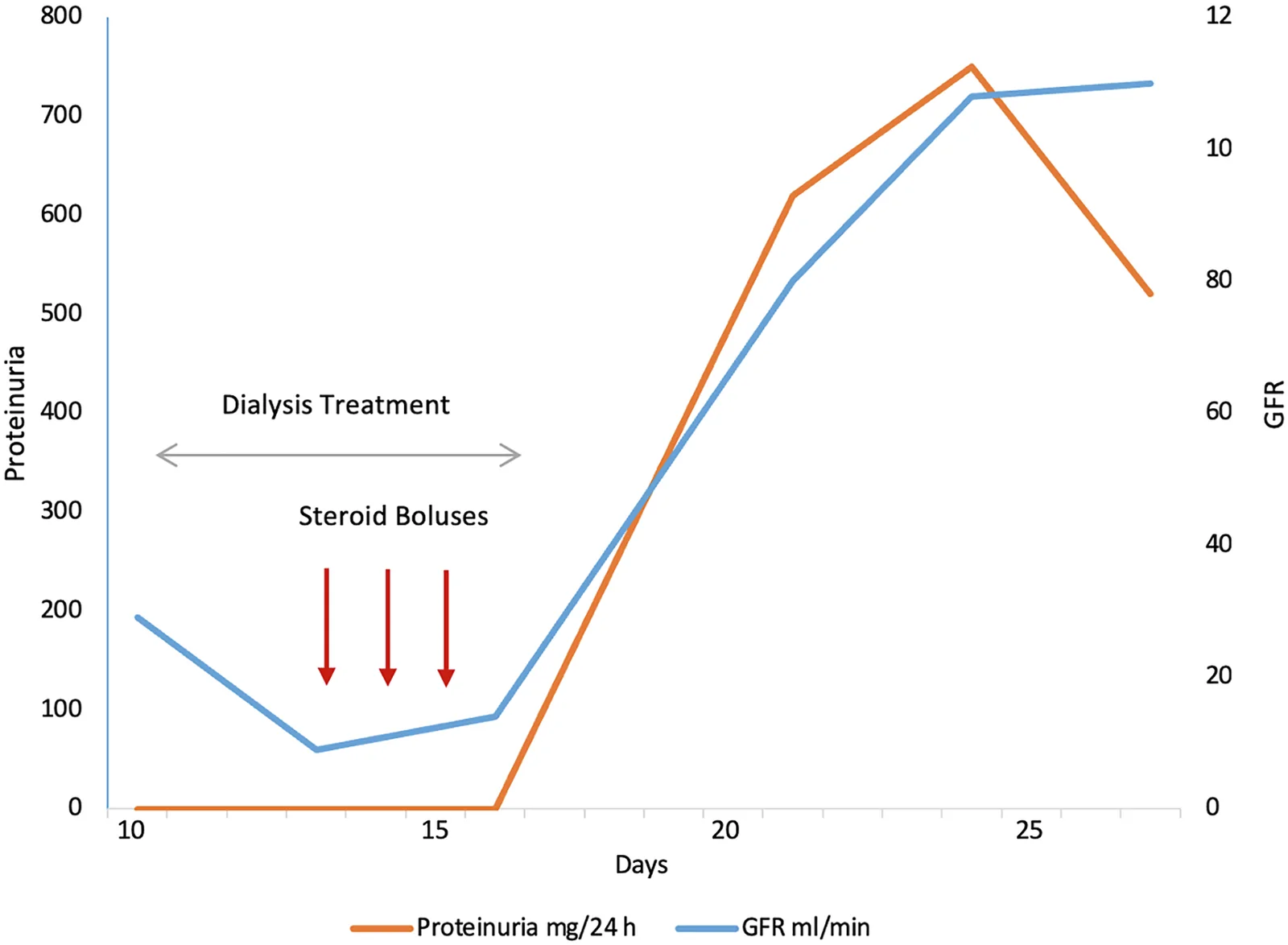
Trend in proteinuria and estimated glomerular filtration rate (GFR) according to CKiDU25 during hospitalization.

Due to persistent oligoanuria (urine output <0.5 mL/kg/h), worsening kidney function (serum creatinine 7.7 mg/dL, serum urea 107 mg/dL, and estimated glomerular filtration rate 8.8 mL/min/1.73 m^2^) and uncontrolled blood pressure (160/90 mm Hg) (Table S5), hemodialysis was started despite stable electrolyte levels (serum sodium 134 mEq/L, serum potassium 4.5 mEq/L). Antihypertensive therapy with a calcium channel blocker and a β-blocker was administered concurrently, although hypertension was likely volume-related (Fig 1). Kidney function gradually improved, accompanied by an increase in 24-hour proteinuria, which peaked at 992 mg/24 h on hospital day 15 (Table S6).

No musculoskeletal, cutaneous, pulmonary, neurologic, or cardiac features suggestive of other systemic vasculitides or rheumatologic disease were identified at clinical or imaging assessment. Kidney ultrasonography revealed kidneys of normal size, echogenicity, and homogeneous parenchymal echotexture, with no evidence of urinary tract dilation.

Laboratory testing revealed normal thyroid function, immunoglobulin (Ig) levels, and coagulation parameters, with negative results for autoimmunity testing (antinuclear antibodies, antineutrophil cytoplasmic antibodies, anti-double-stranded DNA antibodies, and anti–glomerular basement membrane antibodies). Eosinophilia and eosinophiluria were absent (Tables S5 and S6). Infectious viral screening for potential mimickers and triggers, including Epstein–Barr virus, cytomegalovirus, parvovirus B19, hepatitis B virus, hepatitis C virus, and human immunodeficiency virus, was negative, in addition to the results of routine microbiological evaluation.

**Figure 2:**
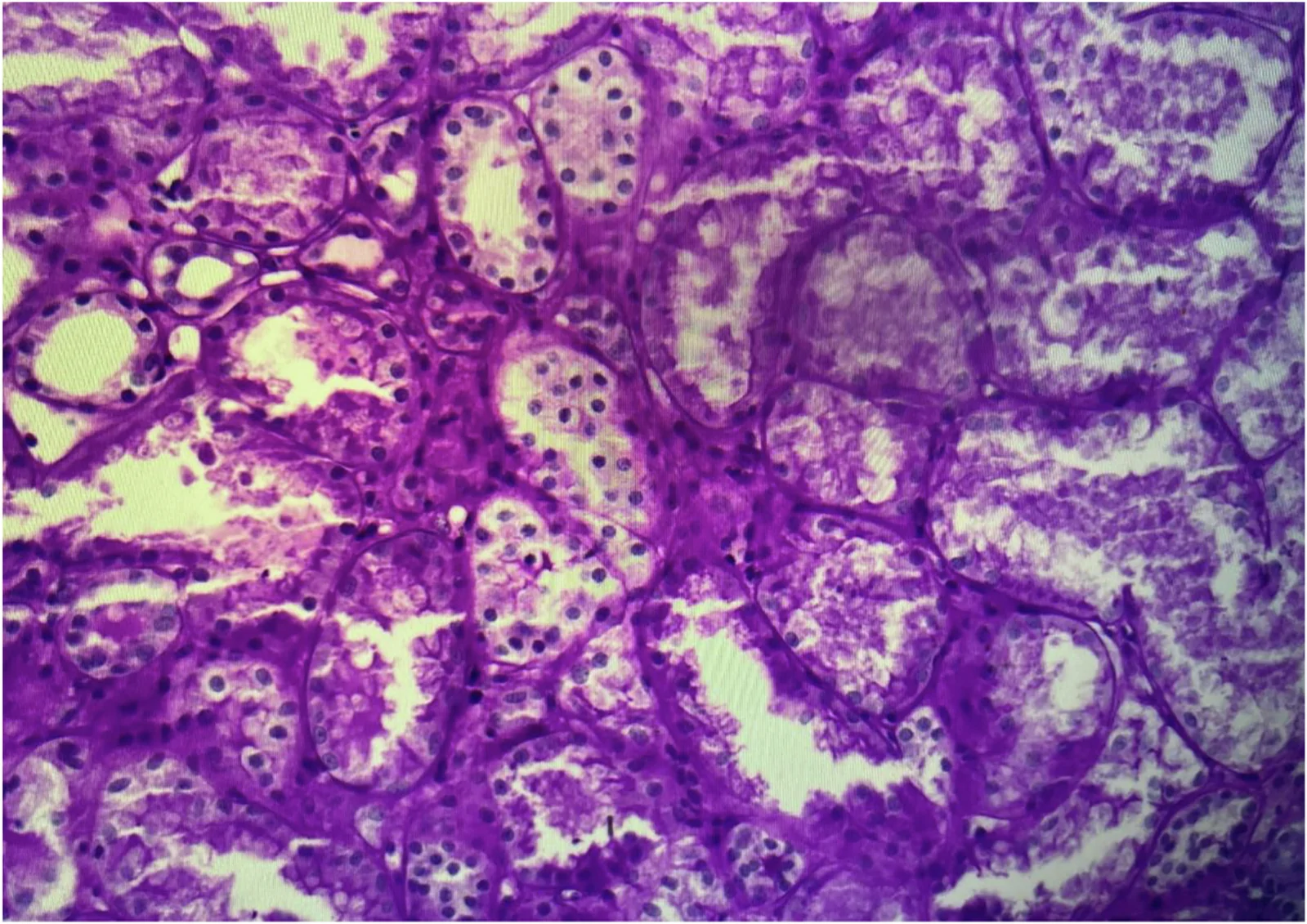
Periodic acid–Schiff staining of a 1-μm semithin resin section showing interstitial inflammation with mild tubular dilation under light microscopy (Original magnification, ×400).

**Figure 3:**
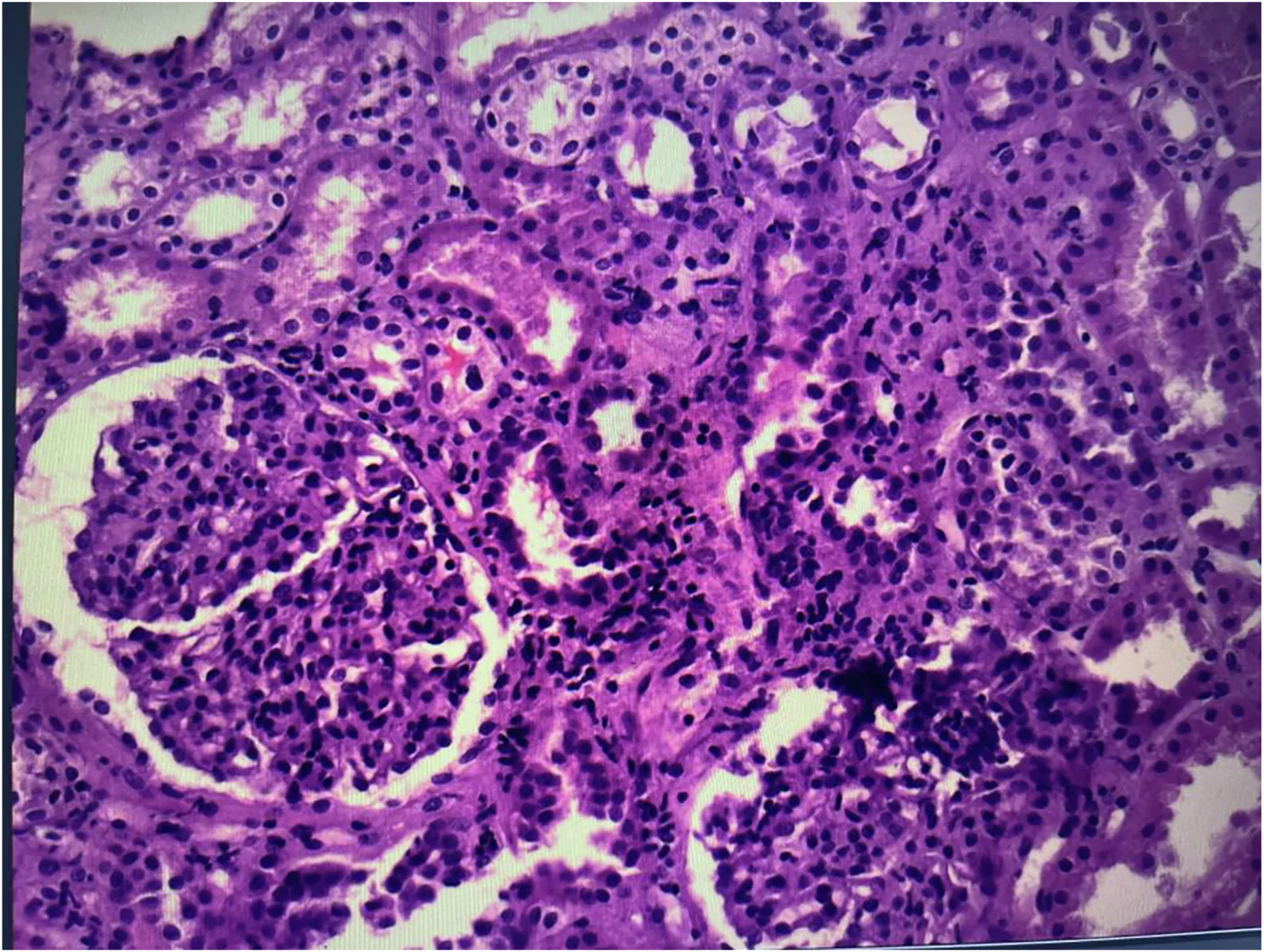
Periodic acid–Schiff staining of a 1-μm semithin resin section showing mesangial hypercellularity and endocapillary cellular proliferation under light microscopy (Original magnification, ×400).

After 5 hemodialysis sessions over 5 days, clinical and hemodynamic stabilization was achieved with a removal of approximately 5 kg of excess fluid. Nevertheless, kidney function remained impaired with oligoanuria. A kidney biopsy was performed, which revealed mild to moderate interstitial inflammation and tubulitis, accompanied by tubular dilation (Fig 2). Focal and mild mesangial hypercellularity was present in 25% of glomeruli, with endocapillary proliferation present in 33% (Fig 3). No evidence of extracapillary proliferation, necrosis, or sclerosis was found. Immunofluorescence showed no glomerular deposits of IgA, IgM, IgG, C3, C4, C1q, κ light chains (κ/λ); however, nonspecific C3 deposits were noted in the tubular epithelium.

The patient was treated with 3 daily intravenous methylprednisolone pulses at 10 mg/kg/d, followed by a 10-week tapering course of oral corticosteroids (Fig 1). Kidney function improved progressively, with recovery of spontaneous diuresis within 8 days. During hospitalization, he had persistent subnephrotic proteinuria (<1 g/24 h), without gross hematuria (Fig 1).

Upon discharge, the patient was in good clinical condition, with an estimated glomerular filtration rate >90 mL/min, and well-controlled blood pressure (135/80 mm Hg) maintained on antihypertensive therapy with amlodipine 5 mg twice daily and carvedilol 6.25 mg twice daily. He was followed up in the nephrology day hospital.

Over the following 10 weeks, proteinuria resolved completely, and antihypertensive medications were discontinued within 3 months.

### Systematic Review

Figure S2 summarizes the results of the literature search and study selection. Among the 8 studies selected, 12 individual patients were identified from 7 case reports (7 patients)^9^, ^10^, ^11^, ^12^, ^13^, ^14^, ^15^ and 1 case series (5 patients).^16^ The geographical distribution of cases was predominantly from the United States (n = 6), followed by Europe (n = 3) and Japan (n = 3).

Table 1 summarizes the main characteristics of included studies. AIN etiologies could be grouped as drug-related, infection-related, and autoimmune-related. Among 9 of the 12 patients with drug-related AIN, antibiotics (eg, cephalosporin, nafcillin)^11^^,^^16^ and NSAIDs (eg, ibuprofen, piroxicam^15^^,^^16^) were the most frequently implicated. Other reported agents included paracetamol,^10^ a diuretic, an H2-blocker (cimetidine),^16^ and an immune checkpoint inhibitor (nivolumab).^13^

**Table 1:** Main characteristics of the eligible studies reporting acute interstitial nephritis overlapping with glomerulonephritis.

Reference, Country	Study	Patients, n	Sex, Age	AIN	Glomerulonephritis	Serology	Treatment	Kidney outcome
Abt^16^ (1985), USA	Case series	5	Case 1: M, 55 y Case 2: F, 70 y Case 3: M, 65 y Case 4: F, 67 y Case 5: F, 31 y	Drug-related (NSAID, diuretic, antibiotic, chemotherapeutic, H2-blocker)	MCD, membranous GN (2), crescentic GN (2)	Negative	Corticosteroids Drug withdrawal	4 complete recoveries 1 partial recovery
Ando^9^ (2013) Japan	Case report	1	M, 20 y	Infection-related (poststreptococcal)	Diffuse proliferative GN	Low complement	Corticosteroids Antibiotics	Complete recovery
Dinesh^12^ (2019), United Kingdom	Case report	1	F, 90 y	Autoimmune-related (IgG4-related disease)	Anti-LRP2 nephropathy with glomerular immune deposits	Negative	Corticosteroids Immunosuppressors	Dialysis-dependent
Feriozzi^11^ (2000), Italy	Case report	1	M, 65 y	Drug-related (cephalosporin)	Pauci-immune GN	MPO-ANCA+	Corticosteroids	Complete recovery
Gallego^10^ (1991), Spain	Case report	1	M, 7 y	Drug-related (paracetamol)	Mesangial GN with C3 deposits	Negative	Drug withdrawal	Complete recovery
Liu^15^ (2021), United States	Case report	1	M, 67 y	Drug-related (NSAID)	MCD	Negative	Corticosteroids Drug withdrawal	Partial recovery
Okunaga^14^ (2023), Japan	Case report	1	M, 78 y	Infection-related (*Staphylococcus aureus*)	IgA-dominant IRGN	Low complement	Corticosteroids Antibiotics	Partial recovery
Tani^13^ (2022), Japan	Case report	1	M, 74 y	Drug-related (nivolumab^a^)	Anti-GBM GN	Anti-GBM+	Corticosteroids Cyclophosphamide Plasmapheresis	Dialysis-dependent

Abbreviations: AIN, acute interstitial nephritis; ANCA, antineutrophil cytoplasmic antibody; GBM, glomerular basement membrane; GN, glomerulonephritis; Ig, immunoglobulin; IRGN, infection-related glomerulonephritis; LRP2, low-density lipoprotein receptor-related protein 2 (megalin); MCD, minimal change disease; MPO, myeloperoxidase. NSAID, non-steroidal anti-inflammatory drug.

a Nivolumab is an immune checkpoint inhibitor.

Clinically, proteinuria was described in 10 of 12 cases, with 6 of 12 patients developing nephrotic-range proteinuria.^9^^,^^10^^,^^14^, ^15^, ^16^ However, the distinction between glomerular and tubular proteinuria was not systematically made. Hematuria occurred in 8 of 12 cases,^9^^,^^10^^,^^12^, ^13^, ^14^, ^15^, ^16^ mostly microscopic (3 of 8),^12^^,^^13^^,^^15^ with only 1 of 8 cases of recurrent gross hematuria.^10^ Systemic hypersensitivity features (rash, fever, or eosinophilia) were reported in 7 of 12 cases.^10^^,^^11^^,^^13^^,^^15^^,^^16^ Cutaneous rash (1 of 12) and fever (3 of 12) were less frequent than eosinophilia in blood or tissue (5 of 12).

Corticosteroids were used in 11 of the 12 cases, regardless of the etiology. In drug-related AIN, corticosteroids were often combined with withdrawal of the offending drug.^10^^,^^11^^,^^13^^,^^15^^,^^16^ Infection-related cases were managed with targeted antibiotics plus corticosteroids.^9^^,^^14^

Regarding kidney outcomes, complete recovery was achieved in 7 of 12 patients,^9^, ^10^, ^11^^,^^14^^,^^16^ partial recovery in 3 of 12 patients,^14^, ^15^, ^16^ and chronic dialysis was required in 2 of 12 patients.^12^^,^^13^

## Discussion

This report describes a probable pediatric case of coexisting IgAV-N and NSAID-induced AIN, illustrating the diagnostic challenge of atypical AKI in systemic vasculitis. Although the initial presentation suggested IgAV-N, the rapid onset of severe oligoanuric stage 3 AKI, with subnephrotic proteinuria and no gross hematuria, was unusual. Kidney biopsy was crucial, showing mild mesangial and endocapillary changes with nonspecific C3 staining, together with interstitial lymphomonocytic infiltrates, tubulitis, and tubular dilatation. The glomerular lesion was considered compatible with IgAV-N but insufficient to explain the severity of AKI, which was more consistent with NSAID-induced AIN. This interpretation was supported by recent high-dose ibuprofen exposure, recovery after drug withdrawal, and absence of active infection. In atypical overlap cases, kidney biopsy is essential to distinguish IgAV-N, AIN, and alternative vasculitic processes. Skin biopsy may be helpful when immunofluorescence is nondiagnostic,^16^ but it was not feasible in this case.

Our systematic review of 12 patients confirms that concurrent AIN and glomerulonephritis, though uncommon, represents an important diagnostic entity. Nearly all published cases involved middle-aged or elderly adults, whereas pediatric cases were rare and largely infection-related.^9^^,^^10^^,^^14^ This makes our case report particularly relevant, suggesting that AIN overlapping with clinically diagnosed IgA vasculitis may be underrecognized in children.

The clinical course in our child was also unusual with only mild proteinuria (<1 g/d) and no gross hematuria, whereas nephrotic-range proteinuria is more often described in glomerulonephritis associated with NSAID-related AIN.^16^

The therapeutic course is likewise notable. Prompt NSAID withdrawal combined with early intravenous corticosteroids led to complete kidney recovery. Only 2 cases in the systematic review required long-term dialysis, although the hemodialysis modality was not specified.^17^ Although steroids are frequently used in drug-induced AIN, evidence on optimal timing, dose, and duration of corticosteroids remains limited.^18^ Corticosteroid use is often guided by clinical uncertainty rather than established benefit. Early steroid treatment may prevent fibrosis by reducing inflammatory infiltrates.^19^

Finally, our case underscores the risk of NSAID use in children. In our child, subclinical glomerular inflammation due to IgAV-N may have amplified NSAID-induced interstitial toxicity. Notably, ibuprofen, an NSAID prescribed to our patient, is the drug most frequently associated with pediatric AIN, especially in infected or dehydrated patients. Limitations of our review are reported in supplementary materials (Item S2: Limitations).

In conclusion, our systematic review shows that concurrent AIN and glomerulonephritis, though uncommon, can manifest with nonspecific AKI, proteinuria, and hematuria. Special caution is warranted when prescribing NSAIDs to pediatric patients, particularly in the presence of underlying glomerular inflammation.

## Supplementary Material

Supplementary File (PDF) Figure S1-S2; Item S1-S2; Figure S1-S2; Table S1-S6.

## References

[1] Davin J.C., Coppo R. (2014). Henoch-Schönlein purpura nephritis in children. Nat Rev Nephrol.

[2] Gardner-Medwin J.M.M., Dolezalova P., Cummins C., Southwood T.R. (2002). Incidence of Henoch-Schönlein purpura, Kawasaki disease, and rare vasculitides in children of different ethnic origins. Lancet.

[3] Sunderkötter C.H., Zelger B., Chen K.R. (2018). Nomenclature of cutaneous vasculitis: dermatologic addendum to the 2012 revised International Chapel Hill Consensus Conference Nomenclature of Vasculitides. Arthritis Rheumatol.

[4] Ozen S., Pistorio A., Iusan S.M., Paediatric Rheumatology International Trials Organisation (PRINTO) (2010). EULAR/PRINTO/PRES criteria for Henoch-Schönlein purpura, childhood polyarteritis nodosa, childhood Wegener granulomatosis and childhood Takayasu arteritis: Ankara 2008. Part II: Final classification criteria. Ann Rheum Dis.

[5] Zhao T., Zhang X., Li Y., Su T. (2025). Durvalumab-associated crescentic glomerulonephritis with IgA vasculitis-like features. Kidney Int Rep.

[6] Kaseda K., Terakawa R., Matsui R. (2025). Successful treatment of MPO-ANCA positive crescentic IgA nephropathy/IgA vasculitis with nephritis potentially triggered by a COVID-19 vaccine in a young adult female using corticosteroids, rituximab, and avacopan. CEN Case Rep.

[7] Kobayashi D., Yoshino J., Hanada M. (2025). A case of de novo glomerulonephritis following COVID-19 in a patient with preexistent IgA vasculitis. CEN Case Rep.

[8] Pierce C.B., Muñoz A., Ng D.K., Warady B.A., Furth S.L., Schwartz G.J. (2021). Age- and sex-dependent clinical equations to estimate glomerular filtration rates in children and young adults with chronic kidney disease. Kidney Int.

[9] Ando F., Sohara E., Ito E. (2013). Acute poststreptococcal glomerulonephritis with acute interstitial nephritis related to streptococcal pyrogenic exotoxin B. Clin Kidney J.

[10] Gallego N., Teruel J.L., Mampaso F., Gonzalo A., Ortuño J. (1991). Acute interstitial nephritis superimposed on glomerulonephritis: report of a case. Pediatr Nephrol.

[11] Feriozzi S., Muda A.O., Gomes V., Montanaro M., Faraggiana T., Ancarani E. (2000). Cephotaxime-associated allergic interstitial nephritis and MPO-ANCA positive vasculitis. Ren Fail.

[12] Dinesh K.P., Raniele D., Michels K. (2019). Anti-LRP2 nephropathy with abundant IgG4-positive plasma cells: a case report. Am J Kidney Dis.

[13] Tani T., Sugino K., Hashimoto K. (2022). Concomitant anti-GBM glomerulonephritis and acute interstitial nephritis following programmed death receptor-1 blockade with nivolumab. Kidney Int Rep.

[14] Okunaga I., Makino S.I., Honda D. (2023). IgA-dominant infection-related glomerulonephritis with NAPlr-positive tubulointerstitial nephritis. CEN Case Rep.

[15] Liu A.C., Chang Y., Zuckerman J.E., Kalantar-Zadeh K., Ghobry L.M., Hanna R.M. (2021). Ibuprofen-associated minimal change disease and acute interstitial nephritis with possibly linked membranous glomerulonephritis. SAGE Open Med Case Rep.

[16] Abt AB, Gordon JA (1985 June). Drug-induced interstitial nephritis. Coexistence with glomerular disease. Arch Intern Med.

[17] Battaglia Y., Shroff R., Meijers B. (2025). Haemodiafiltration versus high-flux haemodialysis—a consensus statement from the EuDial Working Group of the ERA. Nephrol Dial Transplant.

[18] Biederman LE, Conces M, Shenoy A (2023). Acute interstitial nephritis in the pediatric population: a review of etiologic associations, histologic findings, and clinical outcome. Pediatr Dev Pathol.

[19] González E., Gutiérrez E., Galeano C., Grupo Madrileño De Nefritis Intersticiales (2008). Early steroid treatment improves the recovery of renal function in patients with drug-induced acute interstitial nephritis. Kidney Int.

