# Supplementary material for: NSAID-Induced Acute Interstitial Nephritis Concurrent With IgA Vasculitis: A Case-Based Systematic Review
Source: Kidney Med. 2026 Jul 10;8(9):101462. doi: 10.1016/j.xkme.2026.101462 (PMC13524691; doi:10.1016/j.xkme.2026.101462)
Supplement: Supplementary file 1 [file mmc1.pdf]

Item S1. Methods

S1.1. Participant

Our study involved a single case presenting in February 2025, from Santobono Hospital, Naples, Italy, diagnosed with IgAV-N that overlapped with AIN. Pathologists and clinicians analysed the patient's detailed kidney pathology and clinical details. We recorded and compared the patient's demographic information, clinical features, laboratory data, kidney pathology, and treatment response with previously published cases. The case report was structured according to the Case Report (CARE) guidelines (1). Adverse drug reaction (ADR) causality was assessed using the Liverpool ADR Causality Assessment Tool (CAT), classifying the relationship as definite, probable, possible, or unlikely (2). Written informed consent for fully anonymised participation and publication was obtained from the patient, consistent with the CARE statement. All procedures were conducted in accordance with the CARE reporting standards and the ethical principles of the Declaration of Helsinki.

S1.2. Protocol registration

The present systematic review was registered in advance on Open Science Frameworks (registration DOI: 10.17605/OSF.IO/RJPBT).

S1.3. Search strategy and selection criteria

We systematically searched four databases (PubMed, Scopus, Google Scholar, and Web of Science) from inception to 31 October 2025 to identify studies reporting cases of acute interstitial nephritis (AIN) or tubulointerstitial nephritis occurring concurrently with glomerular pathology in adults and children.

The complete Boolean search strings used for each database are reported in **Table S2**. Searches were limited to studies published in the English language. In addition, the reference lists of all included articles and relevant reviews were manually screened to identify any additional eligible studies not captured by the initial search. This systematic review was conducted in accordance with the updated *Preferred Reporting Items for Systematic Reviews and Meta-Analyses* (PRISMA 2020) statement (3). Exclusion criteria included: (i) conference abstracts and practice guidelines; and (ii) studies that did not provide clinical, histological, or therapeutic data relevant to the review objectives.

S1.4. Data extraction and quality assessment

Data from eligible studies were independently extracted by two authors (C.S. and Y.B.). Disagreements at this level were resolved by consensus, with a third author consulted if needed (A.M.). For all studies, we extracted data on the first author, publication year, study country, sample size, population

characteristics, aetiology, serologic parameters, treatment, and outcome. Specifically, the outcomes of interest were complete or partial recovery of kidney function and the need for dialysis. We did not contact any corresponding authors of the included studies to obtain additional information. Due to heterogeneity and the predominance of case reports, a quantitative meta-analysis was not feasible. A PRISMA flow diagram was generated.

Two investigators (YB and CS) independently assessed the methodological quality of all included case reports and case series using the Joanna Briggs Institute (JBI) Critical Appraisal Checklists for Case Reports and Case Series, respectively (4) (**Table S3 and Table S4**). The case-report checklist covers: (a) patient demographics; (b) patient history and timeline; (c) clinical condition at presentation; (d) diagnostic tests/assessment methods and results; (e) interventions; (f) post-intervention clinical condition; (g) adverse or unanticipated events; and (h) key takeaway lessons. The case-series checklist covers: (I) clearly defined inclusion criteria; (II) standard, reliable measurement of the condition; (III) valid methods for case identification; (IV) consecutive inclusion; (V) complete inclusion; (VI) reporting of participant demographics; (VII) reporting of clinical information; (VIII) clarity of outcomes/follow-up; (IX) description of the presenting site(s)/clinic(s); and (X) appropriateness of statistical analysis. Each item was rated Yes, No, Unclear, or Not applicable. Disagreements were resolved by discussion, with arbitration by a third reviewer (AM) if needed.

Supplementary References:

1. Available online on https://www.care-statement.org.

2. Gallagher RM, Kirkham JJ, Mason JR, Bird KA, Williamson PR, Nunn AJ, et al. Development and Inter-Rater Reliability of the Liverpool Adverse Drug Reaction Causality Assessment Tool. Timmer A, editor. PLoS ONE. 2011 Dec 14;6(12):e28096.

3. Page MJ, McKenzie JE, Bossuyt PM, Boutron I, Hoffmann TC, Mulrow CD, Shamseer L, Tetzlaff JM, Akl EA, Brennan SE, Chou R, Glanville J, Grimshaw JM, Hróbjartsson A, Lalu MM, Li T, Loder EW, Mayo-Wilson E, McDonald S, McGuinness LA, Stewart LA, Thomas J, Tricco AC, Welch VA, Whiting P, Moher D. The PRISMA 2020 statement: an updated guideline for reporting systematic reviews. BMJ. 2021 Mar 29;372:n71. doi: 10.1136/bmj.n71.

4. Available online on https://jbi.global/critical-appraisal-tools .

Item S2. Limitations

Firstly, evidence comes almost exclusively from case reports, small series and adults, making it highly susceptible to publication bias. Secondly, the small sample size limits the generalizability of our findings. Thirdly, inconsistent reporting of clinical, laboratory, and outcome data reduces comparability. Finally, the lack of standardised biopsy protocols hinders the evaluation of treatment efficacy on kidney outcomes. Prospective registries are needed to better define pathophysiology, prognosis, and optimal management strategies

Figure S1. Reticular purpuric lesions are confluent on the lower limbs.

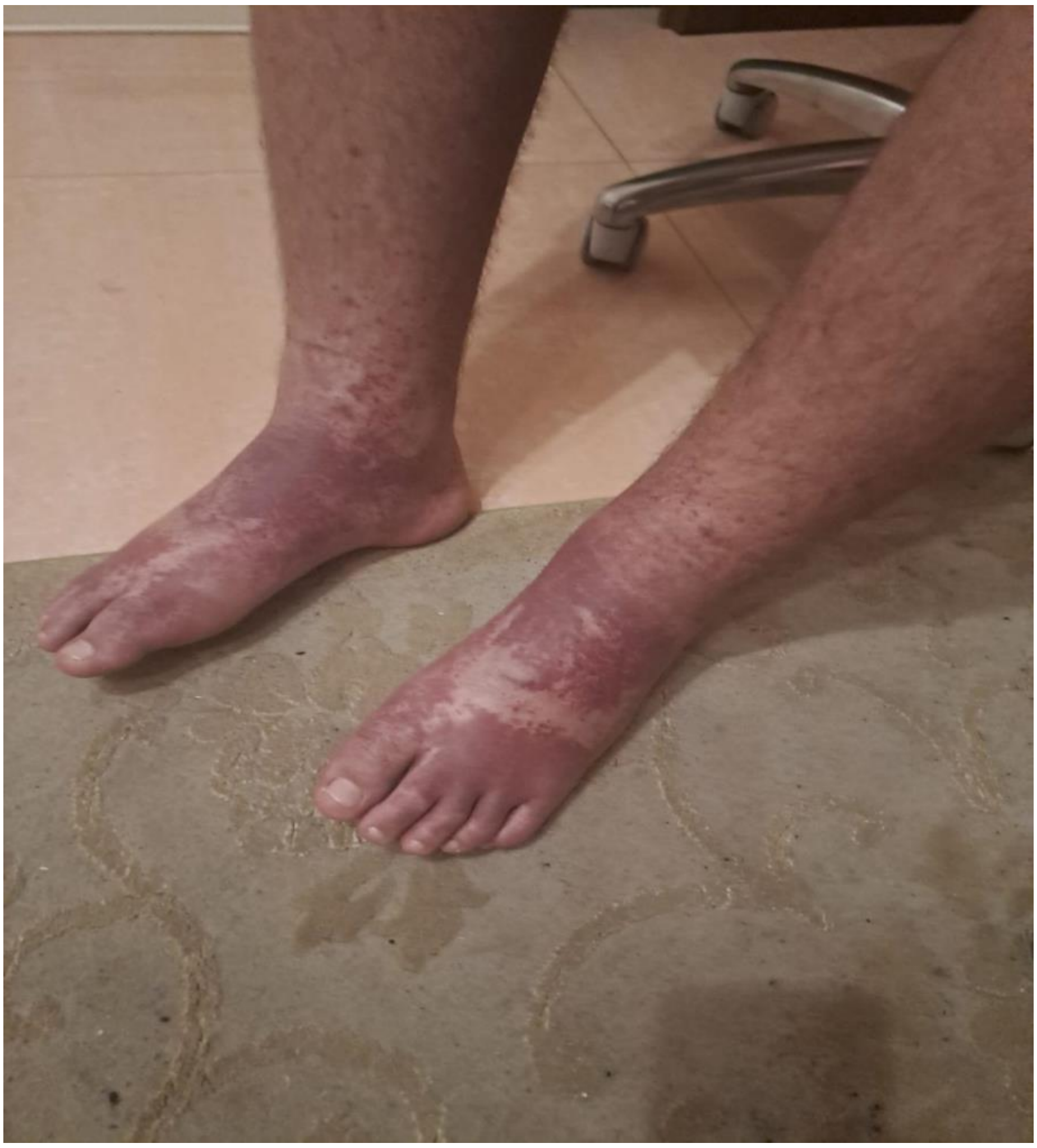

Figure S2. PRISMA flow diagram

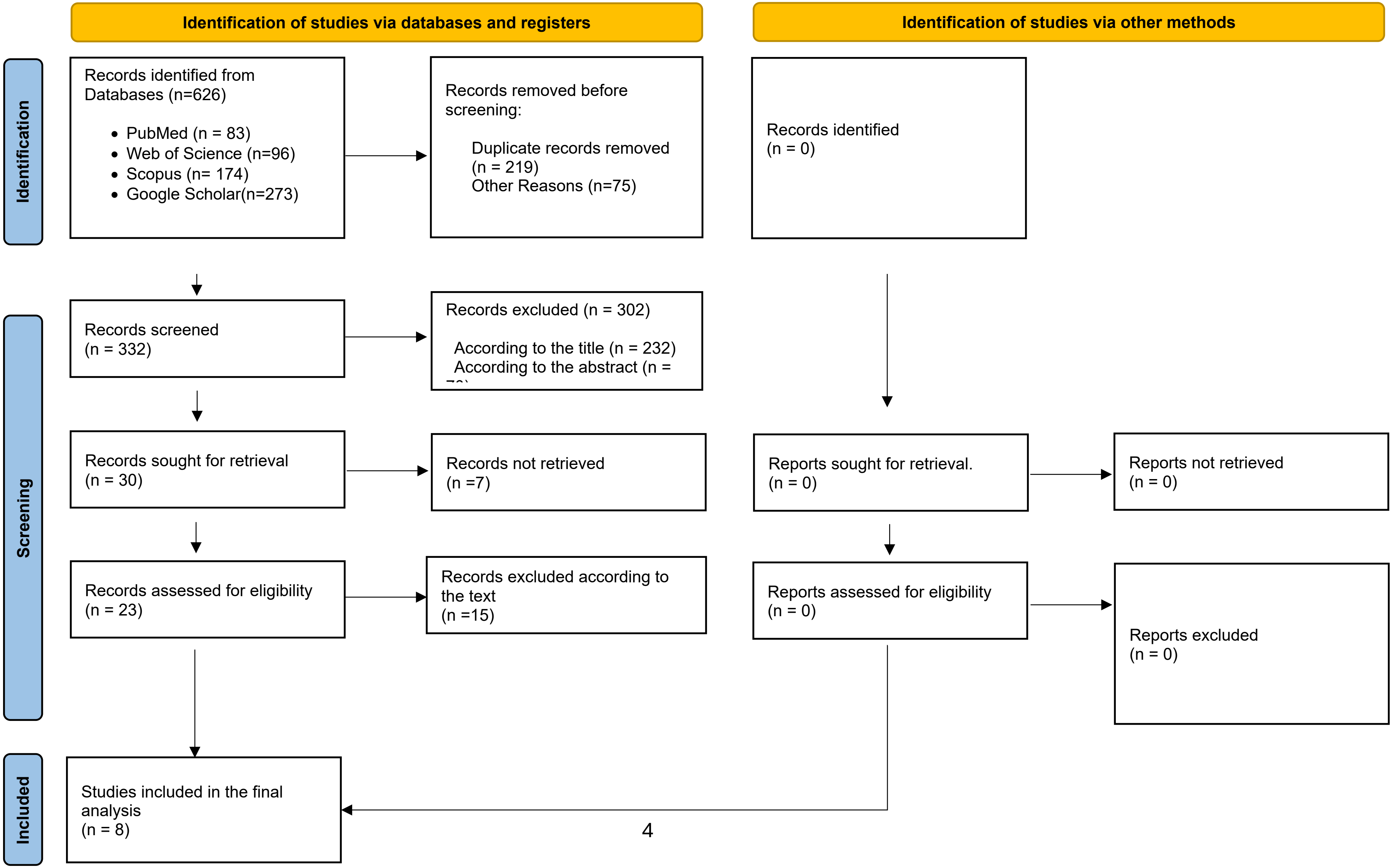

Table S1. Comparative Features of IgA Vasculitis (IgAV-N) and Nonsteroidal Anti-Inflammatory Drugs (NSAIDs) Induced Acute Interstitial Nephritis (AKI).

	**IgAV-N**	**NSAIDs-induced AKI**
**Etiology**	IgA-induced systemic vasculitis	Nephrotoxic effect of NSAIDs
**Age**	Children	Adults
**Trigger**	Respiratory infections,<br>Medications	Prolonged use of NSAIDs
**Pathogenesis**	IgA deposits in small vessels<br>(glomeruli, skin, intestine)	Afferent arteriole vasoconstriction<br>Acute Tubular Necrosis<br>Acute Interstitial Nephritis
**Kidney Manifestation**	Hematuria (micro or macro)<br>Proteinuria<br>Nephritic or nephrotic syndrome	Oliguria<br>Increased creatinine or < GFR<br>Proteinuria < 1 g
**Extrarenal symptoms**	Purpura<br>Arthralgia<br>Abdominal pain	Fever<br>Arthralgia<br>Myalgia<br>Maculopapular skin rashes
**Laboratory tests**	↓ GFR<br>↑ IgA<br>Hematuria<br>Proteinuria	↓↓ GFR<br>↑↑ BUN<br>Eosinophilia (20-40%)
**Urinary sediment**	Red blood cell cylinders<br>Dysmorphic red blood cells	Leukocyturia<br>Hematuria,

		Proteinuria < 1 g/day, (tubular type)
**Kidney Biopsy**	Mesangial Glomerulonephritis	Tubulointerstitial damage
	IgA deposits	Acute tubular necrosis (+/-)
**Therapy**	Corticosteroids	NSAIDs suspension
	Immunosuppressants	Corticosteroids (?)

GFR: glomerular filtration rate

Table S2. Search strings used for each electronic database (last search: October 2025)

DATABASE	SEARCH STRING
**PUBMED**	(("acute interstitial nephritis"[Title/Abstract] OR "tubulointerstitial nephritis"[Title/Abstract]) AND ("glomerulonephritis"[Title/Abstract] OR "ANCA-associated vasculitis"[Title/Abstract] OR "anti-GBM"[Title/Abstract] OR "IgG4-related disease"[Title/Abstract] OR "membranous nephropathy"[Title/Abstract] OR "minimal change disease"[Title/Abstract] OR "infection-related glomerulonephritis"[Title/Abstract]) AND ("drug-induced"[Title/Abstract] OR "NSAID"[Title/Abstract] OR "nonsteroidal anti-inflammatory"[Title/Abstract] OR "immune checkpoint inhibitor"[Title/Abstract] OR "vaccination"[Title/Abstract]))
**SCOPUS**	(TITLE-ABS-KEY("acute interstitial nephritis" OR "tubulointerstitial nephritis")) AND (TITLE-ABS-KEY("glomerulonephritis" OR "ANCA-associated vasculitis" OR "anti-GBM" OR "IgG4-related disease" OR "membranous nephropathy" OR "minimal change disease" OR "infection-related glomerulonephritis" OR "postinfectious glomerulonephritis")) AND (TITLE-ABS-KEY("drug-induced" OR "NSAID" OR "nonsteroidal anti-inflammatory" OR "immune checkpoint inhibitor" OR "vaccination" OR "cefotaxime" OR "paracetamol" OR "ibuprofen" OR "streptococcal" OR "nivolumab"))
**WEB OF SCIENCE**	TS=("acute interstitial nephritis" OR "tubulointerstitial nephritis") AND ("glomerulonephritis" OR "ANCA-associated vasculitis" OR "anti-GBM" OR "IgG4-related disease" OR "membranous nephropathy" OR "minimal change disease" OR "infection-related glomerulonephritis") AND ("drug-induced" OR "NSAID" OR "nonsteroidal anti-inflammatory" OR "immune checkpoint inhibitor" OR "vaccination")
**GOOGLE SCHOLAR**	("acute interstitial nephritis" OR "tubulointerstitial nephritis") AND ("ANCA vasculitis" OR "IgA-dominant" OR "LRP2 nephropathy") AND ("cefotaxime" OR "infection-related" OR "IgG4-positive")

Table S3. Risk of bias assessed by the Joanna Briggs Institute (JBI) Critical Appraisal Checklist for Case Report.

**Authors (Year), Country** (REF)	**Were the patient's demographic characteristics clearly described?**	**Was the patient's history clearly described and presented as a timeline?**	**Was the current clinical condition of the patient on presentation clearly described?**	**Were diagnostic tests or assessment methods and the results clearly described?**	**Was the intervention(s) or treatment procedure(s) clearly described?**	**Was the post-intervention clinical condition clearly described?**	**Were adverse events (harms) or unanticipated events identified and described?**	**Does the case report provide takeaway lessons?**
**Ando (2013), Japan** (18)	yes	yes	yes	yes	yes	yes	yes	yes
**Dinesh (2019), UK** (21)	yes	yes	yes	yes	yes	yes	no	yes
**Feriozzi (2000), Italy** (20)	yes	yes	yes	yes	yes	yes	unclear	yes
**Gallego (1991), Spain** (19)	yes	yes	yes	yes	yes	yes	yes	yes
**Liu (2021), USA** (24)	yes	yes	yes	yes	yes	yes	unclear	yes
**Okunaga (2023), Japan** (23)	yes	yes	yes	yes	yes	yes	unclear	yes
**Tani (2022), Japan** (22)	yes	yes	yes	yes	yes	yes	no	yes

Table S4. Risk of bias assessed by the Joanna Briggs Institute (JBI) Critical Appraisal Checklist for Case Series

**Authors (Year), Country** (REF)	**Were there clear criteria for inclusion in the case series?**	**Was the condition measured in a standard, reliable way for all participants included in the case series?**	**Were valid methods used for the identification of the condition for all participants included in the case series?**	**Did the case series have consecutive inclusion of participants?**	**Did the case series have complete inclusion of participants?**	**Was there clear reporting of the demographics of the participants in the study?**	**Was there clear reporting of clinical information of the participants?**	**Were the outcomes or follow-up results of cases clearly reported?**	**Was there clear reporting of the presenting site(s)/clinic(s) demographic information?**	**Was statistical analysis appropriate?**
**Abt (1985), USA** (25)	yes	yes	yes	no	unclear	yes	yes	yes	yes	Not applicable

Table S5. Trends in Biochemical, Haematological, and Inflammatory Markers.

Parameters	Baseline	Day 6	Day 7	Day 10	Day 12	Day 15	Day 20
Serum Creatinine, mg/dL	0.60	3.04	4.17	7.71	5.08	1.67	1.03
Serum Urea, mg/dL	23	104	107	107	145	139	72
Serum Uric acid, mg/dL	4	8.4	7.2	7	6	8.5	4.8
Serum Glucose mg/dL	78	79	104	137	147	77	76
Serum Sodium mEq/L	138	134	133	134	137	137	135
Serum Potassium mEq/L	4.2	4.2	4.2	4.5	3.8	4.6	4.5
Serum Calcium, mg/dL	8.2	8.6	8.4	9.0	8.2	8.9	9.1
Serum Phosphorus, mg/dL	4.5	4.9	5.7	5.8	5.8	2.5	3.5
Total Cholesterol, mg/dL	115	151	141	175	163	165	164
HDL Cholesterol, mg/dL	29	31	41	38	44	38	48
Triglycerides, mg/dL	105	136	111	98	86	85	149
AST UI/L	35	39	26	28	23	25	11
ALT UI/L	33	30	24	13	17	18	14
LDH UI/L	396	457	481	396	263	333	258
Red cells, 10^6/mL	8880	4.45	4.15	4.20	3.79	3.90	4.02
Haemoglobin, g/dL	12.7	12.2	11.3	10.8	10.5	11.5	11.2
WBC, 10^3/mcL	8.880	11.56	9.86	12.05	12.17	15.75	16.05
PLT, 10^3/mcL	224	209	163	350	333	454	416
CRP, mg/L	45.10	18.6	55.2	46.65	12.34	1.16	0.5
PCT, ng/mL	0.18	0.13	0.25	0.71	0.25	0.04	0.04
Serum Iron, mg/dL		48	33	86	118	181	159

AST: Aspartate Aminotransferase, ALT: Alanine Aminotransferase, CRP: C-Reactive Protein, C3: Complement Component 3, C4: Complement Component 4, LDH: Lactate Dehydrogenase, PCT: Procalcitonin, PLT: Platelets, TAS: Total Antioxidant Status, WBC: White blood cells.

Table S6. Trends in Urinalysis and 24-Hour Urine Protein.

**Parameters**	**Baseline**	**Day 6**	**Day 7**	**Day 10**	**Day 12**	**Day 15**	**Day 20**	**Day 21**
pH	5.5	5.0	5.0	5	5.5	5.5	6	6
Specific weight	1039	1046	1033	1019	1021	1021	1018	1018
Haemoglobin, mg/dL	0,03	0.1	0.2	0.1	0.1	0.1	0	0
Urine protein, mg/dL	>30				28	55	22	23
Urine protein, mg/24-h					620	992	428	531
Urinary creatinine, mg/24-h					83.2	95	91.2	79.2
Urinary creatinine, mg/24-h					1830	1709	1732	1820
Urinary protein/creatinine ratio, mg/g					0.34	0.58	0.25	0.29
